# *P*-[*N*-(Diphenyl­phospho­rothio­yl)iso­propyl­amino]-*N*-isopropyl-*P*-phenyl­thio­phosphinic amide

**DOI:** 10.1107/S1600536809014238

**Published:** 2009-04-22

**Authors:** Normen Peulecke, Bhaskar R. Aluri, Anina Wöhl, Anke Spannenberg, Mohammed H. Al-Hazmi

**Affiliations:** a Leibniz-Institut für Katalyse e. V. an der Universität Rostock, Albert-Einstein-Strasse 29a, 18059 Rostock, Germany; b Linde AG, Linde Engineering Division, Dr-Carl-von-Linde-Strasse 6-14, 82049 Pullach, Germany; c Sabic R&T Complex, Catalysis and Specialty Section, Chemical Research Department, PO Box 42503, Riyadh 11551, Saudi Arabia

## Abstract

The title compound, C_24_H_30_N_2_P_2_S_2_, was obtained by the reaction of Ph_2_PN(*i*Pr)P(Ph)N(*i*Pr)H with elemental sulfur in tetra­hydro­furan. In the solid state, intra­molecular N—H⋯S hydrogen bonding influences the mol­ecular conformation; a P—N—P—N torsion angle of 2.28 (9)° is observed. The two phenyl rings attached to one P atom form a dihedral angle of 74.02 (4)°.

## Related literature

For the crystal structures of similar compounds, see: Alouani *et al.* (2007 ▶); Bent *et al.* (1990 ▶); Simón-Manso *et al.* (2002 ▶); Ziegler & Weiss (1968 ▶). Synthesis of the starting compound Ph_2_PN(*i*Pr)P(Ph)N(*i*Pr)H was reported by Müller *et al.* (2009 ▶).
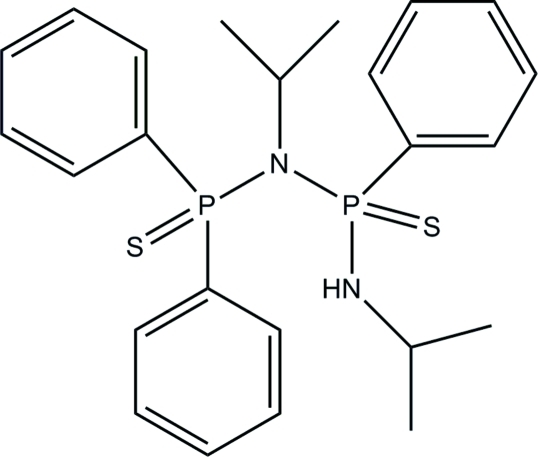


## Experimental

### 

#### Crystal data

C_24_H_30_N_2_P_2_S_2_
*M* *_r_* = 472.56
Monoclinic, 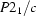

*a* = 9.08354 (19) Å
*b* = 25.4654 (7) Å
*c* = 10.6557 (2) Å
β = 100.1488 (17)°
*V* = 2426.26 (10) Å^3^
*Z* = 4
Mo *K*α radiation
μ = 0.37 mm^−1^
*T* = 200 K
0.45 × 0.25 × 0.20 mm

#### Data collection

Stoe IPDS-II diffractometer
Absorption correction: numerical (*X-SHAPE*; Stoe & Cie, 2005 ▶) *T*_min_ = 0.835, *T*_max_ = 0.954
40233 measured reflections
5561 independent reflections
4459 reflections with *I* > 2σ(*I*)
*R*_int_ = 0.032

#### Refinement

*R*[*F* ^2^ > 2σ(*F* ^2^)] = 0.028
*wR*(*F* ^2^) = 0.074
*S* = 1.02
5561 reflections
275 parameters
H atoms treated by a mixture of independent and constrained refinement
Δρ_max_ = 0.21 e Å^−3^
Δρ_min_ = −0.29 e Å^−3^

### 

Data collection: *X-AREA* (Stoe & Cie, 2005 ▶); cell refinement: *X-AREA*; data reduction: *X-RED* (Stoe & Cie, 2005 ▶); program(s) used to solve structure: *SHELXS97* (Sheldrick, 2008 ▶); program(s) used to refine structure: *SHELXL97* (Sheldrick, 2008 ▶); molecular graphics: *XP* in *SHELXTL* (Sheldrick, 2008 ▶); software used to prepare material for publication: *SHELXTL*.

## Supplementary Material

Crystal structure: contains datablocks I, global. DOI: 10.1107/S1600536809014238/cv2547sup1.cif

Structure factors: contains datablocks I. DOI: 10.1107/S1600536809014238/cv2547Isup2.hkl

Additional supplementary materials:  crystallographic information; 3D view; checkCIF report

## Figures and Tables

**Table 1:** Hydrogen-bond geometry (Å, °)

*D*—H⋯*A*	*D*—H	H⋯*A*	*D*⋯*A*	*D*—H⋯*A*
N2—H2⋯S1	0.860 (19)	2.578 (19)	3.2963 (12)	141.7 (16)

**Fig. 1.**
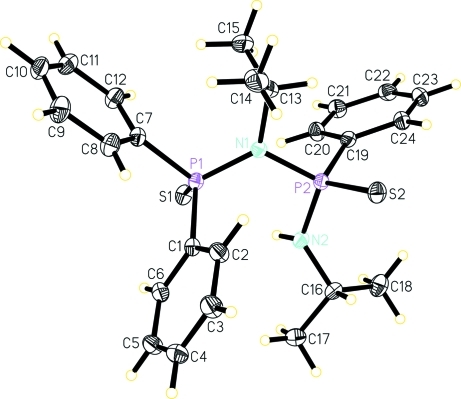
The molecular structure of the title compound showing the labelling scheme. Atomic displacement ellipsoids are drawn at the 30% probability level.

## supplementary crystallographic information

### Comment

Linear phosphazanes can act as chelate ligands containing both hard (nitrogen) and soft (phosphorus) donor atoms. Often these compounds are thermally unstable and undergo rapid oxidation. Crystal structures of the compounds with a P(S)–N–P(S) unit are already known *e.g.* [(Me_2_N)_2_P(S)]_2_NMe (Alouani *et al.*, 2007), C_6_H_4_(NH)P(S)EtNP(S)(NEt_2_)Et (Bent *et al.*, 1990), [Ph_2_P(S)]_2_N(CHMePh) (Simón-Manso *et al.*, 2002) and NH_2_(NHMe)P(S)N(Me)P(S)(NH_2_)_2_ (Ziegler *et al.*, 1968). In the present publication, we report on the formation and molecular structure of C_24_H_30_N_2_P_2_S_2_, which was observed to be the single product of a complete oxidation of Ph_2_PN(*i*Pr)P(Ph)N(*i*Pr)H with sulfur. The starting compound was synthesized as described in the patent WO 2009006979 (Müller *et al.*, 2009).

In the solid state a torsion angle P1—N1—P2—N2 of 2.28 (9)° was found for the title compound. The two phenyl rings attached to P1 form a dihedral angle of 74.02 (4)°. A weak intramolecular hydrogen bond N2—H2···S1 (Table 1) was observed.

### Experimental

204 mg (0,5 mmol) Ph_2_PN(*i*Pr)P(Ph)N(*i*Pr)H and 38.5 mg (1.2 mmol) sulfur were solved in 10 ml tetrahydrofuran and stirred for 24 h at 40°C. The solution was filtrated to remove unreacted sulfur. The major part of tetrahydrofuran was removed and the remaining solution was over-layered with *n*-hexane to get single crystals of the title compound, which are suitable for X-ray analysis. The white compound was fully characterized by standard analytical methods *e.g.*^31^P NMR: (C_6_D_6_): 73.7, 65.9 (broad).

### Refinement

Atom H2 attached to N2 was found on a difference Fourier map and refined isotropically. All other H atoms were placed in idealized positions with d(C—H) = 0.98 (CH_3_) and 0.95–1.00 Å (CH) and refined using a riding model with *U*_iso_(H) fixed at 1.5 *U*_eq_(C) for CH_3_ and 1.2 *U*_eq_(C) for CH.

### Crystal data

**Table:**

C_24_H_30_N_2_P_2_S_2_	*F*(000) = 1000
*M**_r_* = 472.56	*D*_x_ = 1.294 Mg m^−^^3^
Monoclinic, *P*2_1_/*c*	Mo *K*α radiation, λ = 0.71073 Å
Hall symbol: -P 2ybc	Cell parameters from 32201 reflections
*a* = 9.08354 (19) Å	θ = 1.6–29.6°
*b* = 25.4654 (7) Å	µ = 0.37 mm^−^^1^
*c* = 10.6557 (2) Å	*T* = 200 K
β = 100.1488 (17)°	Prism, colourless
*V* = 2426.26 (10) Å^3^	0.45 × 0.25 × 0.20 mm
*Z* = 4	

### Data collection

**Table:**

Stoe IPDS-II diffractometer	5561 independent reflections
Radiation source: fine-focus sealed tube	4459 reflections with *I* > 2σ(*I*)
graphite	*R*_int_ = 0.032
ω scans	θ_max_ = 27.5°, θ_min_ = 1.6°
Absorption correction: numerical (*X-SHAPE*; Stoe & Cie, 2005)	*h* = −11→11
*T*_min_ = 0.835, *T*_max_ = 0.954	*k* = −32→32
40233 measured reflections	*l* = −13→13

### Refinement

**Table:**

Refinement on *F*^2^	Primary atom site location: structure-invariant direct methods
Least-squares matrix: full	Secondary atom site location: difference Fourier map
*R*[*F*^2^ > 2σ(*F*^2^)] = 0.028	Hydrogen site location: inferred from neighbouring sites
*wR*(*F*^2^) = 0.074	H atoms treated by a mixture of independent and constrained refinement
*S* = 1.02	*w* = 1/[σ^2^(*F*_o_^2^) + (0.0472*P*)^2^] where *P* = (*F*_o_^2^ + 2*F*_c_^2^)/3
5561 reflections	(Δ/σ)_max_ = 0.001
275 parameters	Δρ_max_ = 0.21 e Å^−^^3^
0 restraints	Δρ_min_ = −0.28 e Å^−^^3^

### Special details

**Table:**

Geometry. All e.s.d.'s (except the e.s.d. in the dihedral angle between two l.s. planes) are estimated using the full covariance matrix. The cell e.s.d.'s are taken into account individually in the estimation of e.s.d.'s in distances, angles and torsion angles; correlations between e.s.d.'s in cell parameters are only used when they are defined by crystal symmetry. An approximate (isotropic) treatment of cell e.s.d.'s is used for estimating e.s.d.'s involving l.s. planes.
Refinement. Refinement of *F*^2^ against ALL reflections. The weighted *R*-factor *wR* and goodness of fit *S* are based on *F*^2^, conventional *R*-factors *R* are based on *F*, with *F* set to zero for negative *F*^2^. The threshold expression of *F*^2^ > σ(*F*^2^) is used only for calculating *R*-factors(gt) *etc*. and is not relevant to the choice of reflections for refinement. *R*-factors based on *F*^2^ are statistically about twice as large as those based on *F*, and *R*- factors based on ALL data will be even larger.

### Fractional atomic coordinates and isotropic or equivalent isotropic displacement parameters (Å^2^)

**Table:**

	*x*	*y*	*z*	*U*_iso_*/*U*_eq_	
C1	0.10362 (14)	0.36285 (5)	0.96160 (12)	0.0273 (3)	
C2	0.03459 (17)	0.41159 (5)	0.96335 (13)	0.0340 (3)	
H2A	−0.0041	0.4289	0.8855	0.041*	
C3	0.02223 (19)	0.43493 (6)	1.07847 (14)	0.0408 (3)	
H3A	−0.0262	0.4680	1.0794	0.049*	
C4	0.0804 (2)	0.41023 (6)	1.19223 (14)	0.0420 (4)	
H4A	0.0722	0.4264	1.2711	0.050*	
C5	0.15020 (18)	0.36216 (6)	1.19100 (14)	0.0390 (3)	
H5A	0.1908	0.3454	1.2691	0.047*	
C6	0.16137 (15)	0.33825 (6)	1.07616 (13)	0.0322 (3)	
H6A	0.2086	0.3050	1.0758	0.039*	
C7	−0.07285 (15)	0.29565 (5)	0.77859 (13)	0.0291 (3)	
C8	−0.19321 (16)	0.31171 (6)	0.83371 (15)	0.0368 (3)	
H8A	−0.1839	0.3417	0.8876	0.044*	
C9	−0.32629 (17)	0.28415 (7)	0.81026 (17)	0.0457 (4)	
H9A	−0.4081	0.2954	0.8482	0.055*	
C10	−0.34149 (18)	0.24077 (7)	0.73275 (16)	0.0444 (4)	
H10A	−0.4343	0.2227	0.7150	0.053*	
C11	−0.22185 (19)	0.22362 (7)	0.68090 (16)	0.0450 (4)	
H11A	−0.2314	0.1932	0.6286	0.054*	
C12	−0.08742 (17)	0.25057 (6)	0.70473 (15)	0.0382 (3)	
H12A	−0.0045	0.2380	0.6701	0.046*	
C13	0.00109 (16)	0.38464 (6)	0.57993 (13)	0.0356 (3)	
H13A	0.0478	0.4120	0.5323	0.043*	
C14	−0.14292 (18)	0.40909 (7)	0.60510 (17)	0.0489 (4)	
H14A	−0.1196	0.4391	0.6628	0.073*	
H14B	−0.1998	0.3830	0.6444	0.073*	
H14C	−0.2026	0.4210	0.5244	0.073*	
C15	−0.0230 (2)	0.33753 (7)	0.49071 (15)	0.0519 (4)	
H15A	0.0741	0.3238	0.4780	0.078*	
H15B	−0.0812	0.3483	0.4084	0.078*	
H15C	−0.0773	0.3101	0.5283	0.078*	
C16	0.47654 (15)	0.43480 (5)	0.92161 (13)	0.0312 (3)	
H16A	0.4224	0.4681	0.9341	0.037*	
C17	0.5216 (2)	0.40897 (7)	1.05016 (16)	0.0526 (5)	
H17A	0.4318	0.4007	1.0856	0.079*	
H17B	0.5854	0.4329	1.1078	0.079*	
H17C	0.5767	0.3766	1.0405	0.079*	
C18	0.61096 (18)	0.44872 (7)	0.86165 (17)	0.0447 (4)	
H18A	0.5771	0.4653	0.7786	0.067*	
H18B	0.6671	0.4167	0.8502	0.067*	
H18C	0.6755	0.4731	0.9174	0.067*	
C19	0.34557 (15)	0.40237 (5)	0.57479 (12)	0.0284 (3)	
C20	0.39580 (17)	0.35128 (6)	0.56205 (13)	0.0353 (3)	
H20A	0.3719	0.3245	0.6171	0.042*	
C21	0.48033 (17)	0.33927 (6)	0.46965 (14)	0.0387 (3)	
H21A	0.5154	0.3045	0.4621	0.046*	
C22	0.51353 (17)	0.37820 (6)	0.38836 (13)	0.0385 (3)	
H22A	0.5725	0.3702	0.3256	0.046*	
C23	0.46149 (18)	0.42831 (6)	0.39823 (14)	0.0411 (4)	
H23A	0.4831	0.4547	0.3410	0.049*	
C24	0.37749 (17)	0.44093 (6)	0.49107 (13)	0.0349 (3)	
H24A	0.3420	0.4758	0.4974	0.042*	
N1	0.11346 (12)	0.37534 (4)	0.70123 (10)	0.0262 (2)	
N2	0.37172 (13)	0.39982 (5)	0.83996 (11)	0.0284 (2)	
H2	0.392 (2)	0.3668 (8)	0.8425 (17)	0.047 (5)*	
P1	0.10723 (4)	0.328578 (13)	0.81322 (3)	0.02526 (8)	
P2	0.25813 (4)	0.418481 (13)	0.71077 (3)	0.02554 (8)	
S1	0.26642 (4)	0.275781 (13)	0.82829 (4)	0.03334 (9)	
S2	0.20212 (4)	0.491907 (14)	0.71375 (3)	0.03440 (9)	

### Atomic displacement parameters (Å^2^)

**Table:**

	*U*^11^	*U*^22^	*U*^33^	*U*^12^	*U*^13^	*U*^23^
C1	0.0269 (6)	0.0265 (6)	0.0292 (6)	−0.0029 (5)	0.0068 (5)	0.0017 (5)
C2	0.0442 (8)	0.0298 (7)	0.0289 (7)	0.0034 (6)	0.0086 (6)	0.0017 (5)
C3	0.0550 (10)	0.0326 (8)	0.0375 (8)	0.0051 (7)	0.0156 (7)	−0.0029 (6)
C4	0.0554 (10)	0.0430 (9)	0.0294 (7)	−0.0052 (7)	0.0122 (7)	−0.0048 (6)
C5	0.0424 (8)	0.0451 (9)	0.0291 (7)	−0.0018 (7)	0.0052 (6)	0.0054 (6)
C6	0.0317 (7)	0.0325 (7)	0.0331 (7)	0.0000 (6)	0.0073 (5)	0.0054 (5)
C7	0.0267 (6)	0.0282 (6)	0.0330 (7)	−0.0016 (5)	0.0072 (5)	0.0023 (5)
C8	0.0300 (7)	0.0338 (7)	0.0485 (8)	0.0001 (6)	0.0119 (6)	−0.0020 (6)
C9	0.0288 (7)	0.0477 (9)	0.0627 (10)	−0.0018 (7)	0.0144 (7)	0.0030 (8)
C10	0.0320 (8)	0.0465 (9)	0.0526 (9)	−0.0124 (7)	0.0017 (7)	0.0090 (7)
C11	0.0451 (9)	0.0409 (9)	0.0476 (9)	−0.0132 (7)	0.0046 (7)	−0.0073 (7)
C12	0.0363 (8)	0.0358 (8)	0.0439 (8)	−0.0054 (6)	0.0113 (6)	−0.0072 (6)
C13	0.0334 (7)	0.0448 (8)	0.0270 (7)	−0.0068 (6)	0.0008 (6)	0.0053 (6)
C14	0.0325 (8)	0.0620 (11)	0.0488 (9)	0.0044 (7)	−0.0019 (7)	0.0148 (8)
C15	0.0611 (11)	0.0634 (11)	0.0297 (7)	−0.0207 (9)	0.0038 (7)	−0.0062 (7)
C16	0.0304 (7)	0.0290 (7)	0.0332 (7)	−0.0036 (5)	0.0029 (5)	−0.0045 (5)
C17	0.0590 (11)	0.0577 (11)	0.0356 (8)	−0.0176 (8)	−0.0068 (7)	0.0018 (7)
C18	0.0335 (8)	0.0423 (9)	0.0588 (10)	−0.0073 (7)	0.0092 (7)	−0.0004 (7)
C19	0.0267 (6)	0.0305 (7)	0.0282 (6)	−0.0042 (5)	0.0052 (5)	−0.0006 (5)
C20	0.0418 (8)	0.0318 (7)	0.0351 (7)	0.0000 (6)	0.0143 (6)	0.0012 (6)
C21	0.0420 (8)	0.0396 (8)	0.0366 (8)	0.0024 (6)	0.0127 (6)	−0.0036 (6)
C22	0.0374 (8)	0.0509 (9)	0.0294 (7)	−0.0050 (7)	0.0117 (6)	−0.0048 (6)
C23	0.0497 (9)	0.0445 (9)	0.0312 (7)	−0.0092 (7)	0.0129 (6)	0.0032 (6)
C24	0.0402 (8)	0.0327 (7)	0.0327 (7)	−0.0038 (6)	0.0086 (6)	0.0026 (5)
N1	0.0245 (5)	0.0288 (6)	0.0253 (5)	−0.0024 (4)	0.0042 (4)	−0.0001 (4)
N2	0.0288 (6)	0.0246 (6)	0.0311 (6)	−0.0022 (4)	0.0035 (5)	−0.0017 (4)
P1	0.02403 (16)	0.02397 (16)	0.02854 (16)	−0.00070 (12)	0.00670 (12)	−0.00037 (12)
P2	0.02616 (16)	0.02422 (16)	0.02674 (16)	−0.00164 (12)	0.00604 (12)	−0.00036 (12)
S1	0.02795 (17)	0.02701 (17)	0.0461 (2)	0.00268 (13)	0.00941 (14)	0.00022 (14)
S2	0.03846 (19)	0.02535 (17)	0.04002 (19)	0.00214 (14)	0.00865 (15)	0.00032 (13)

### Geometric parameters (Å, °)

**Table:**

C1—C6	1.3900 (18)	C15—H15A	0.9800
C1—C2	1.3922 (19)	C15—H15B	0.9800
C1—P1	1.8113 (13)	C15—H15C	0.9800
C2—C3	1.385 (2)	C16—N2	1.4714 (17)
C2—H2A	0.9500	C16—C17	1.510 (2)
C3—C4	1.385 (2)	C16—C18	1.516 (2)
C3—H3A	0.9500	C16—H16A	1.0000
C4—C5	1.380 (2)	C17—H17A	0.9800
C4—H4A	0.9500	C17—H17B	0.9800
C5—C6	1.386 (2)	C17—H17C	0.9800
C5—H5A	0.9500	C18—H18A	0.9800
C6—H6A	0.9500	C18—H18B	0.9800
C7—C12	1.385 (2)	C18—H18C	0.9800
C7—C8	1.391 (2)	C19—C24	1.3911 (19)
C7—P1	1.8173 (14)	C19—C20	1.393 (2)
C8—C9	1.382 (2)	C19—P2	1.8178 (14)
C8—H8A	0.9500	C20—C21	1.385 (2)
C9—C10	1.372 (2)	C20—H20A	0.9500
C9—H9A	0.9500	C21—C22	1.384 (2)
C10—C11	1.374 (2)	C21—H21A	0.9500
C10—H10A	0.9500	C22—C23	1.371 (2)
C11—C12	1.385 (2)	C22—H22A	0.9500
C11—H11A	0.9500	C23—C24	1.389 (2)
C12—H12A	0.9500	C23—H23A	0.9500
C13—C14	1.515 (2)	C24—H24A	0.9500
C13—N1	1.5177 (16)	N1—P1	1.6938 (11)
C13—C15	1.522 (2)	N1—P2	1.7021 (11)
C13—H13A	1.0000	N2—P2	1.6386 (12)
C14—H14A	0.9800	N2—H2	0.860 (19)
C14—H14B	0.9800	P1—S1	1.9602 (5)
C14—H14C	0.9800	P2—S2	1.9395 (5)
			
C6—C1—C2	119.42 (12)	N2—C16—C17	108.44 (12)
C6—C1—P1	119.16 (10)	N2—C16—C18	112.18 (12)
C2—C1—P1	121.25 (10)	C17—C16—C18	112.00 (13)
C3—C2—C1	120.10 (13)	N2—C16—H16A	108.0
C3—C2—H2A	120.0	C17—C16—H16A	108.0
C1—C2—H2A	120.0	C18—C16—H16A	108.0
C4—C3—C2	120.14 (14)	C16—C17—H17A	109.5
C4—C3—H3A	119.9	C16—C17—H17B	109.5
C2—C3—H3A	119.9	H17A—C17—H17B	109.5
C5—C4—C3	119.97 (14)	C16—C17—H17C	109.5
C5—C4—H4A	120.0	H17A—C17—H17C	109.5
C3—C4—H4A	120.0	H17B—C17—H17C	109.5
C4—C5—C6	120.22 (14)	C16—C18—H18A	109.5
C4—C5—H5A	119.9	C16—C18—H18B	109.5
C6—C5—H5A	119.9	H18A—C18—H18B	109.5
C5—C6—C1	120.14 (13)	C16—C18—H18C	109.5
C5—C6—H6A	119.9	H18A—C18—H18C	109.5
C1—C6—H6A	119.9	H18B—C18—H18C	109.5
C12—C7—C8	118.70 (13)	C24—C19—C20	119.20 (13)
C12—C7—P1	119.36 (11)	C24—C19—P2	121.44 (11)
C8—C7—P1	121.67 (11)	C20—C19—P2	119.01 (10)
C9—C8—C7	120.14 (14)	C21—C20—C19	120.49 (13)
C9—C8—H8A	119.9	C21—C20—H20A	119.8
C7—C8—H8A	119.9	C19—C20—H20A	119.8
C10—C9—C8	120.65 (15)	C22—C21—C20	119.73 (15)
C10—C9—H9A	119.7	C22—C21—H21A	120.1
C8—C9—H9A	119.7	C20—C21—H21A	120.1
C9—C10—C11	119.69 (14)	C23—C22—C21	120.16 (14)
C9—C10—H10A	120.2	C23—C22—H22A	119.9
C11—C10—H10A	120.2	C21—C22—H22A	119.9
C10—C11—C12	120.20 (15)	C22—C23—C24	120.66 (14)
C10—C11—H11A	119.9	C22—C23—H23A	119.7
C12—C11—H11A	119.9	C24—C23—H23A	119.7
C11—C12—C7	120.53 (14)	C23—C24—C19	119.74 (14)
C11—C12—H12A	119.7	C23—C24—H24A	120.1
C7—C12—H12A	119.7	C19—C24—H24A	120.1
C14—C13—N1	112.68 (12)	C13—N1—P1	127.50 (9)
C14—C13—C15	113.63 (14)	C13—N1—P2	110.27 (8)
N1—C13—C15	114.19 (13)	P1—N1—P2	122.20 (6)
C14—C13—H13A	105.1	C16—N2—P2	124.56 (10)
N1—C13—H13A	105.1	C16—N2—H2	117.2 (12)
C15—C13—H13A	105.1	P2—N2—H2	114.3 (12)
C13—C14—H14A	109.5	N1—P1—C1	106.51 (6)
C13—C14—H14B	109.5	N1—P1—C7	108.78 (6)
H14A—C14—H14B	109.5	C1—P1—C7	104.21 (6)
C13—C14—H14C	109.5	N1—P1—S1	115.10 (4)
H14A—C14—H14C	109.5	C1—P1—S1	112.68 (5)
H14B—C14—H14C	109.5	C7—P1—S1	108.95 (5)
C13—C15—H15A	109.5	N2—P2—N1	103.20 (6)
C13—C15—H15B	109.5	N2—P2—C19	107.78 (6)
H15A—C15—H15B	109.5	N1—P2—C19	104.35 (6)
C13—C15—H15C	109.5	N2—P2—S2	113.21 (5)
H15A—C15—H15C	109.5	N1—P2—S2	114.92 (4)
H15B—C15—H15C	109.5	C19—P2—S2	112.54 (5)
			
C6—C1—C2—C3	−0.8 (2)	C13—N1—P1—C7	−8.37 (13)
P1—C1—C2—C3	174.52 (12)	P2—N1—P1—C7	173.69 (7)
C1—C2—C3—C4	0.9 (2)	C13—N1—P1—S1	114.18 (11)
C2—C3—C4—C5	−0.3 (3)	P2—N1—P1—S1	−63.76 (8)
C3—C4—C5—C6	−0.5 (2)	C6—C1—P1—N1	−152.53 (10)
C4—C5—C6—C1	0.7 (2)	C2—C1—P1—N1	32.18 (13)
C2—C1—C6—C5	0.0 (2)	C6—C1—P1—C7	92.55 (11)
P1—C1—C6—C5	−175.41 (11)	C2—C1—P1—C7	−82.74 (12)
C12—C7—C8—C9	−2.7 (2)	C6—C1—P1—S1	−25.41 (12)
P1—C7—C8—C9	−176.74 (12)	C2—C1—P1—S1	159.29 (10)
C7—C8—C9—C10	0.0 (3)	C12—C7—P1—N1	92.52 (12)
C8—C9—C10—C11	1.9 (3)	C8—C7—P1—N1	−93.43 (13)
C9—C10—C11—C12	−1.3 (3)	C12—C7—P1—C1	−154.16 (12)
C10—C11—C12—C7	−1.4 (2)	C8—C7—P1—C1	19.89 (13)
C8—C7—C12—C11	3.3 (2)	C12—C7—P1—S1	−33.67 (13)
P1—C7—C12—C11	177.57 (12)	C8—C7—P1—S1	140.39 (11)
C24—C19—C20—C21	1.9 (2)	C16—N2—P2—N1	−152.15 (11)
P2—C19—C20—C21	−171.38 (11)	C16—N2—P2—C19	97.82 (12)
C19—C20—C21—C22	−0.8 (2)	C16—N2—P2—S2	−27.31 (12)
C20—C21—C22—C23	−0.8 (2)	C13—N1—P2—N2	−175.97 (9)
C21—C22—C23—C24	1.2 (2)	P1—N1—P2—N2	2.28 (9)
C22—C23—C24—C19	−0.1 (2)	C13—N1—P2—C19	−63.41 (10)
C20—C19—C24—C23	−1.5 (2)	P1—N1—P2—C19	114.84 (8)
P2—C19—C24—C23	171.67 (11)	C13—N1—P2—S2	60.30 (10)
C14—C13—N1—P1	73.52 (16)	P1—N1—P2—S2	−121.44 (6)
C15—C13—N1—P1	−58.09 (17)	C24—C19—P2—N2	−120.52 (12)
C14—C13—N1—P2	−108.34 (12)	C20—C19—P2—N2	52.61 (13)
C15—C13—N1—P2	120.05 (12)	C24—C19—P2—N1	130.24 (11)
C17—C16—N2—P2	161.08 (12)	C20—C19—P2—N1	−56.62 (12)
C18—C16—N2—P2	−74.69 (15)	C24—C19—P2—S2	5.00 (13)
C13—N1—P1—C1	−120.16 (12)	C20—C19—P2—S2	178.14 (10)
P2—N1—P1—C1	61.90 (9)		

### Hydrogen-bond geometry (Å, °)

**Table:**

*D*—H···*A*	*D*—H	H···*A*	*D*···*A*	*D*—H···*A*
N2—H2···S1	0.860 (19)	2.578 (19)	3.2963 (12)	141.7 (16)

## References

[bb1] Alouani, K., Raouafi, N. & Guesmi, A. (2007). *Struct. Chem.* **18**, 569–572.

[bb2] Bent, E. G., Schaeffer, R., Haltiwanger, R. C. & Norman, A. D. (1990). *Inorg. Chem.* **29**, 2608–2613.

[bb3] Müller, B. H., Fritz, P., Bölt, H., Wöhl, A., Müller, W., Winkler, F., Wellenhofer, A., Rosenthal, U., Hapke, M., Peulecke, N., Al-Hazmi, M. H., Aliyev, V. O. & Mosa, F. M. (2009). WO Patent No. 2009006979. (Linde AG, Saudi Basic Industries Corporation, January 15, 2009.)

[bb4] Sheldrick, G. M. (2008). *Acta Cryst.* A**64**, 112–122. 10.1107/S0108767307043930 18156677

[bb5] Simón-Manso, E., Valderrama, M., Gantzel, P. & Kubiak, C. P. (2002). *J. Organomet. Chem.* **651**, 90–97.

[bb6] Stoe & Cie (2005). *X-SHAPE*, *X-RED* and *X-AREA* Stoe & Cie, Darmstadt, Germany.

[bb7] Ziegler, M. L. & Weiss, J. (1968). *Z. Anorg. Allg. Chem.* **361**, 136–146.

